# Where it all started

**DOI:** 10.7554/eLife.87355

**Published:** 2023-04-05

**Authors:** Long Zhang, Na Li, Ren Xu

**Affiliations:** 1 The First Affiliated Hospital of Xiamen University‐ICMRS Collaborating Center for Skeletal Stem Cell, State Key Laboratory of Cellular Stress Biology, Faculty of Medicine and Life Sciences, Xiamen University Xiamen China; 2 Fujian Provincial Key Laboratory of Organ and Tissue Regeneration, School of Medicine, Xiamen University Xiamen China

**Keywords:** articular cartilage, articular chondrocyte, progenitors, differentiation, transcriptional factor, NFAT, Mouse

## Abstract

The progenitor cells that form articular cartilage express a gene for a protein called NFATc1, which stops articular chondrocytes from developing too early in the joint.

**Related research article** Zhang F, Wang Y, Zhao Y, Wang M, Zhou B, Zhou B, Ge X. 2023. NFATc1 marks articular cartilage progenitors and negatively determines articular chondrocyte differentiation. *eLife* **12**:e81569. doi: 10.7554/eLife.81569.

Articular cartilage is a smooth white tissue that covers the ends of bones where they join together. It is essential for maintaining the mobility of bone joints. However, despite this important role, the tissue is susceptible to degeneration caused by trauma, disease or ageing. This can lead to conditions such as osteoarthritis, which can cause chronic pain and, in some cases, disability (Cui et al., 2020).

Reversing damage to articular cartilage remains a great challenge in musculoskeletal medicine. One way to tackle this issue is to better understand how articular cartilage forms during embryonic development, as this knowledge could help researchers to develop new methods for rebuilding cartilage. Yet, the origins of the main cells in cartilage, known as articular chondrocytes, and the identity of the genes that regulate their production remain unclear. Now, in eLife, Xianpeng Ge (Capital Medical University in Beijing) and colleagues – including Fan Zhang and Yuanyuan Wang as joint first authors – report new insights into the formation of articular chondrocytes (Zhang et al., 2023).

Previous research has shown that the gene *NFATc1* helps to regulate the development of bones by controlling cells that absorb and form bone (Winslow et al., 2006). To find out if *NFATc1* could also be involved in cartilage development, Zhang et al. used genetically modified mouse embryos, in which cells expressing *NFATc1* were tagged with a fluorescent marker that allowed the researchers to track these cells and their offspring over time. This revealed that *NFATc1* is expressed in a group of progenitor cells throughout embryonic and even postnatal development, and that these cells are responsible for generating most of the articular chondrocytes (Figure 1).

**Figure 1.**
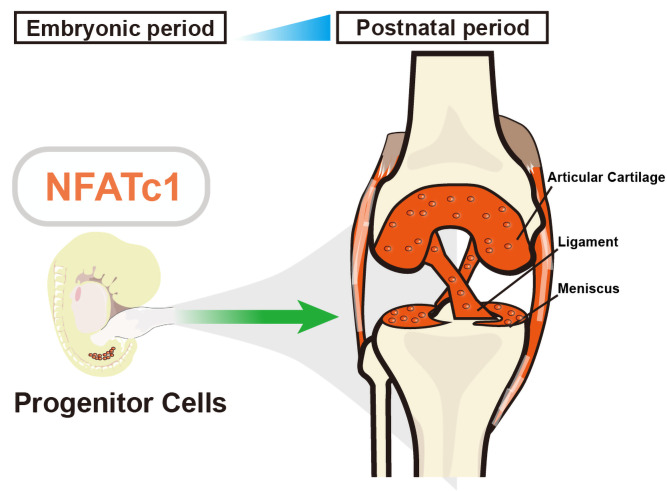
The role of NFATc1 in articular cartilage formation. Zhang et al. identified a unique progenitor cell population in the knee joint of embryonic mice (left) that expresses a gene called *NFATc1*, which has previously been linked to bone formation and bone resorption. These progenitor (stem) cells are responsible for generating articular chondrocytes – the main cell type in articular cartilage – during both embryonic and postnatal development (right). Suppressing the activity of *NFATc1* in these stem progenitor cells induces the formation of chondrocytes, which are essential for forming articular cartilage.

Further experiments in cells and embryonic mice revealed that the expression of *NFATc1* decreased as the articular chondrocytes matured. Zhang et al. found that when the gene was removed from the cartilage progenitors, the maturation of chondrocytes was faster and the formation of articular cartilage in embryos was still supported. Consistent with this, when *NFATc1* was overactivated, the progenitor cells could no longer mature into chondrocytes. This indicates that this gene negatively regulates the development of articular chondrocytes.

So far, it has been challenging to track how articular cartilage develops as most genetic markers cannot distinguish between the different cell types in the relevant tissues (Rux et al., 2019; Chijimatsu and Saito, 2019). The only exception is the gene *Prg4*, a widely used marker of articular cartilage progenitors. But since cells only start to express this gene at a later stage during development, the marker fails to track their movement during the earlier stages (Kozhemyakina et al., 2015). In contrast, *NFATc1* is expressed early during articular cartilage development, and may therefore offer a better alternative for studying the origin of articular cartilage.

It was previously thought that *NFATc1*, together with another gene involved in cartilage formation, *NFATc2*, restricts the overgrowth of cartilage (also known as osteochondroma) at the sites where the ligaments insert into the bone. However, adjusting how much of either of those genes was removed from the mice’s progenitor cells could in fact determine the number and size of osteochondromas (Ge et al., 2016). This knowledge may be helpful in understanding how *NFATc1* may suppress the formation of cartilage in certain diseases.

It has been suggested that both *NFATc1* and *NFATc2* are key suppressors of osteoarthritis, which is crucial for articular cartilage formation in mice (Greenblatt et al., 2013). However, when Zhang et al. used a different approach to delete these two genes, they observed the opposite effect – removing *NFATc1* and *NFATc2* enhanced chondrogenesis under pathological conditions. This suggests that researchers should carefully consider the genetic ablating tools they use when studying the role of progenitor cells in articular cartilage.

Moreover, it has been shown that adult skeletal stem cells expressing *NFATc1* behave as bone-cell progenitors and contribute to the early stages of repair following a bone fracture (Yu et al., 2022). This highlights the complex pool of progenitor cells that express *NFATc1*, and how the fate of these progenitors changes during different periods of development as well as adulthood. More importantly, to date, it remains unclear whether these *NFATc1*-expressing progenitor cells fulfill stemness criteria in the skeleton, such as stem-cell transplantation (Debnath et al., 2018), or if these cells are able to generate functional articular cartilage.

Despite a lack of evidence confirming the stemness of *NFATc1*-expressing progenitor cells, the work of Zhang et al. offers researchers an additional genetic tool to detect and track the origins of articular chondrocytes. Moreover, their findings provide valuable insights into the mechanism of articular cartilage formation. If studies in human cells achieve similar results, this may help scientists to identify potential treatments of cartilage diseases, such as osteoarthritis.

## References

[bib1] Chijimatsu R, Saito T (2019). Mechanisms of synovial joint and articular cartilage development. Cellular and Molecular Life Sciences.

[bib2] Cui A, Li H, Wang D, Zhong J, Chen Y, Lu H (2020). Global, regional prevalence, incidence and risk factors of knee osteoarthritis in population-based studies. EClinicalMedicine.

[bib3] Debnath S, Yallowitz AR, McCormick J, Lalani S, Zhang T, Xu R, Li N, Liu Y, Yang YS, Eiseman M, Shim JH, Hameed M, Healey JH, Bostrom MP, Landau DA, Greenblatt MB (2018). Discovery of a periosteal stem cell mediating intramembranous bone formation. Nature.

[bib4] Ge X, Tsang K, He L, Garcia RA, Ermann J, Mizoguchi F, Zhang M, Zhou B, Zhou B, Aliprantis AO (2016). NFAT restricts osteochondroma formation from entheseal progenitors. JCI Insight.

[bib5] Greenblatt MB, Ritter SY, Wright J, Tsang K, Hu D, Glimcher LH, Aliprantis AO (2013). NFATc1 and NFATc2 repress spontaneous osteoarthritis. PNAS.

[bib6] Kozhemyakina E, Zhang M, Ionescu A, Ayturk UM, Ono N, Kobayashi A, Kronenberg H, Warman ML, Lassar AB (2015). Identification of a *Prg4*-expressing articular cartilage progenitor cell population in mice. Arthritis & Rheumatology.

[bib7] Rux D, Decker RS, Koyama E, Pacifici M (2019). Joints in the appendicular skeleton: developmental mechanisms and evolutionary influences. Current Topics in Developmental Biology.

[bib8] Winslow MM, Pan M, Starbuck M, Gallo EM, Deng L, Karsenty G, Crabtree GR (2006). Calcineurin/NFAT signaling in osteoblasts regulates bone mass. Developmental Cell.

[bib9] Yu F, Li F, Yu P, Zhou B, Ye L (2022). Identification and characterization of NFATc1+ skeletal stem cells in bone regeneration. Cell Reports.

[bib10] Zhang F, Wang Y, Zhao Y, Wang M, Zhou B, Zhou B, Ge X (2023). NFATc1 marks articular cartilage progenitors and negatively determines articular chondrocyte differentiation. eLife.

